# Alteration of Transcripts of Stress-Protective Genes and Transcriptional Factors by γ-Aminobutyric Acid (GABA) Associated with Improved Heat and Drought Tolerance in Creeping Bentgrass (*Agrostis stolonifera*)

**DOI:** 10.3390/ijms19061623

**Published:** 2018-05-31

**Authors:** Zhou Li, Yan Peng, Bingru Huang

**Affiliations:** 1 Department of Grassland Science, College of Animal Science and Technology, Sichuan Agricultural University, Chengdu 611130, Sichuan, China; lizhou1986814@163.com (Z.L.); pengyanlee@163.com (Y.P.); 2 Department of Plant Biology and Pathology, Rutgers University, 59 Dudley Road, New Brunswick, NJ 08901, USA

**Keywords:** high temperature, water stress, dehydrins, heat shock protein, antioxidant genes, plant growth regulator

## Abstract

Gamma-aminobutyric acid (GABA) may play a positive role in regulating plant tolerance to drought or heat stress. The objectives of this study were to investigate the physiological effects of GABA on tolerance of creeping bentgrass (*Agrostis stolonifera*) to heat and drought stress and to determine whether enhanced heat and drought tolerance due to GABA treatment was associated with the up-regulation of selected genes and transcriptional factors involved in stress protection. Creeping bentgrass (cultivar “Penncross”) plants were treated with 0.5 mM GABA or water (untreated control) as a foliar spray and were subsequently exposed to heat stress (35/30 °C, day/night), drought stress by withholding irrigation, or non-stress conditions in controlled-environment growth chambers. Exogenous application of GABA significantly improved plant tolerance to heat and drought stress, as reflected by increased leaf water content, cell membrane stability, and chlorophyll content. The analysis of gene transcript level revealed that exogenous GABA up-regulated the expression of *ABF3*, *POD*, *APX*, *HSP90*, *DHN3*, and *MT1* during heat stress and the expression of *CDPK26*, *MAPK1*, *ABF3*, *WRKY75*, *MYB13*, *HSP70*, *MT1*, *14-3-3*, and genes (*SOD*, *CAT*, *POD*, *APX*, *MDHAR*, *DHAR*, and *GR*) encoding antioxidant enzymes during drought stress. The up-regulation of the aforementioned stress-protective genes and transcriptional factors could contribute to improved heat and drought tolerance in creeping bentgrass.

## 1. Introduction

Abiotic stresses such as heat and drought stress limit plant growth in many areas of the world [1,2]. Heat and drought damages typically are associated with growth inhibition, oxidative damage due to accumulation of reactive oxygen species (ROS), and accelerated leaf senescence [3,4,5]. Plants have developed multiple adaptive mechanisms such as the activation of stress-protective genes encoding heat shock proteins (HSPs), dehydrins (DHNs), metallothionein (MT), and proteins involved in antioxidant metabolism, as well as many transcriptional factors, such as the *WRKY*, *MYB*, and *bZIP* family [6,7,8,9,10]. Approaches such as exogenous application of plant growth regulators (PGR) that can induce or enhance the expression of stress-protective genes and transcriptional factors are found to be effective in improving tolerance to heat and drought stress in perennial grass and other plant species [9,11,12].

Previous studies showed that γ-aminobutyric acid (GABA), known as a non-protein amino acid, exhibits plant growth regulator (PGR) properties and plays a role in the regulation of plant tolerance to abiotic stresses [13]. The accumulation of GABA was found in various plant species such as Bermuda grass (*Cynodon dactylon*), pea (*Pisum sativum*), and *Caragana intermedia* in response to heat, drought, and salt stress [14,15,16]. It has also been reported that exogenous application of GABA could improve heat tolerance in rice (*Oryza sativa*) and drought tolerance in black pepper (*Piper nigrum*) [17,18]. In contrast, the GABA-depleted *Arabidopsis* mutant wilted earlier than the wild type during prolonged drought stress [19]. GABA was shown to have multiple functions involved in regulation of proline and tricarboxylic acid (TCA) cycle metabolism, mitigation of oxidative damage, and maintenance of carbon and nitrogen metabolism associated with tolerance to abiotic stresses in plant species [20,21,22]. GABA was also involved in the regulation of the process of signaling transduction and might be considered as a signal molecule for triggering downstream gene expression [23,24]. Currently, mechanisms of how GABA may lead to enhanced heat and drought tolerance are still not well understood, despite there being known positive physiological effects associated with the compound. As discussed above, many stress-related genes have been identified in plants associated with signal transduction, transcription factors, and genes encoding antioxidant enzymes and stress-induced proteins and their transcript levels are positively associated with enhanced stress tolerance [9,10,25]. However, limited information is available regarding common or specific genes’ expression regulated by GABA that may be associated with improved heat and drought tolerance when plants are subjected to these stresses.

The objectives of the present study were to investigate the physiological effects of GABA on heat and drought tolerance for creeping bentgrass, a cool-season perennial grass species, and to determine whether enhanced heat and drought tolerance conferred by GABA was associated with the up-regulation of genes involved in stress protection. The findings of this study will provide a better understanding of defense mechanisms activated by GABA at the molecular level when plants respond to different abiotic stresses. Creeping bentgrass is a cool-season grass species adapted to cold and humid climates and is sensitive to drought and high temperature but due to its superior quality as turfgrass, its use has been expanded widely to warm climate regions; however, high temperature and drought have become the main abiotic stress factors leading to the decline in turf quality and increased maintenance costs of creeping bentgrass, especially in the summer [26,27]. The information obtained from this study also has practical implications of improving heat and drought tolerance through genetic modification of GABA-regulated genes and exogenous application of GABA for cool-season turfgrass species in dry and hot environments.

## 2. Results

### 2.1. Physiological Effects of GABA under Heat and Drought Stress

**Figure 1:**
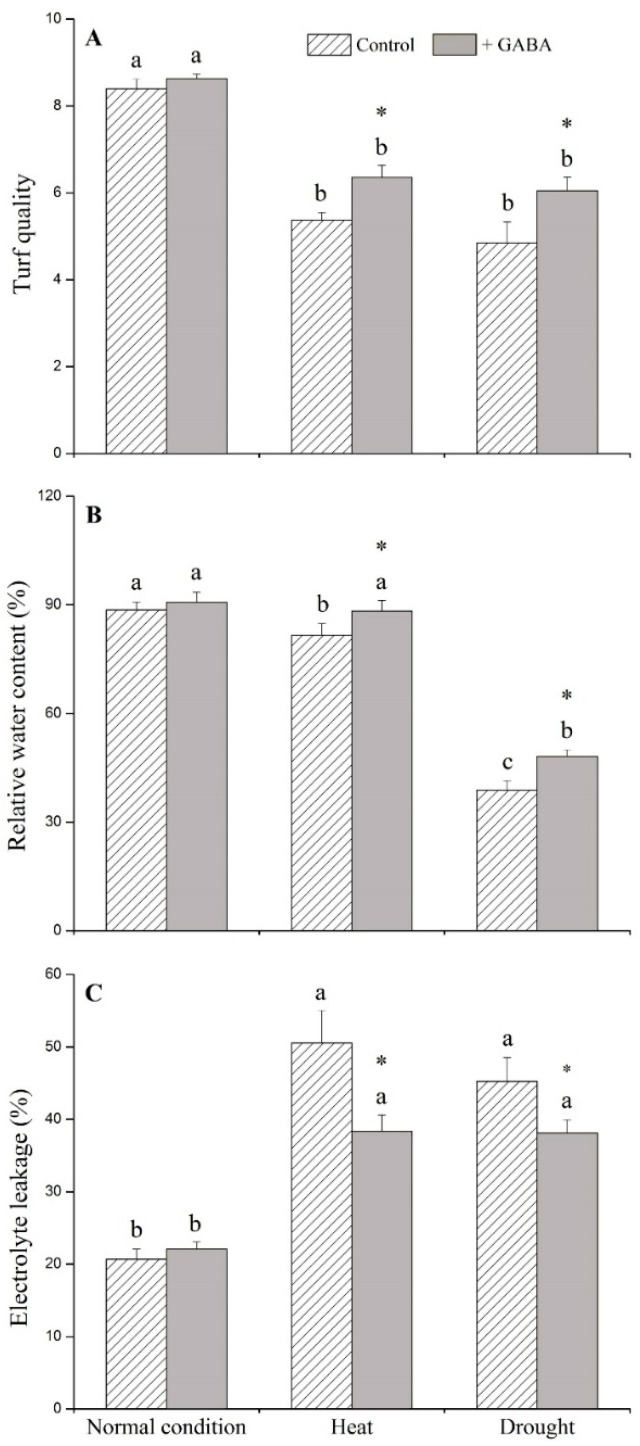
Effects of γ-aminobutyric acid (GABA) on (**A**) turf quality (TQ), (**B**) relative water content (RWC), and (**C**) electrolyte leakage (EL) in creeping bentgrass under normal, heat, and drought conditions. Vertical bars indicate ±standard error (SE) of mean (*n* = 4). Different letters above columns indicate significant differences for one particular treatment (control or +GABA) under different conditions; asterisk “*” indicates significant difference between control and +GABA treatment under a given condition.

**Figure 2:**
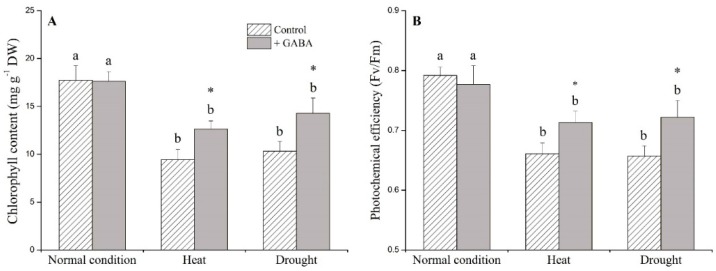
Effects of γ-aminobutyric acid (GABA) on (**A**) chlorophyll content (Chl) and (**B**) photochemical efficiency (Fv/Fm) in creeping bentgrass under normal, heat, and drought condition. Vertical bars indicate ±SE of mean (*n* = 4). Different letters above columns indicate significant differences for one particular treatment (control or +GABA) under different conditions; asterisk “*” indicates significant difference between control and +GABA treatment under a given condition.

**Figure 3:**
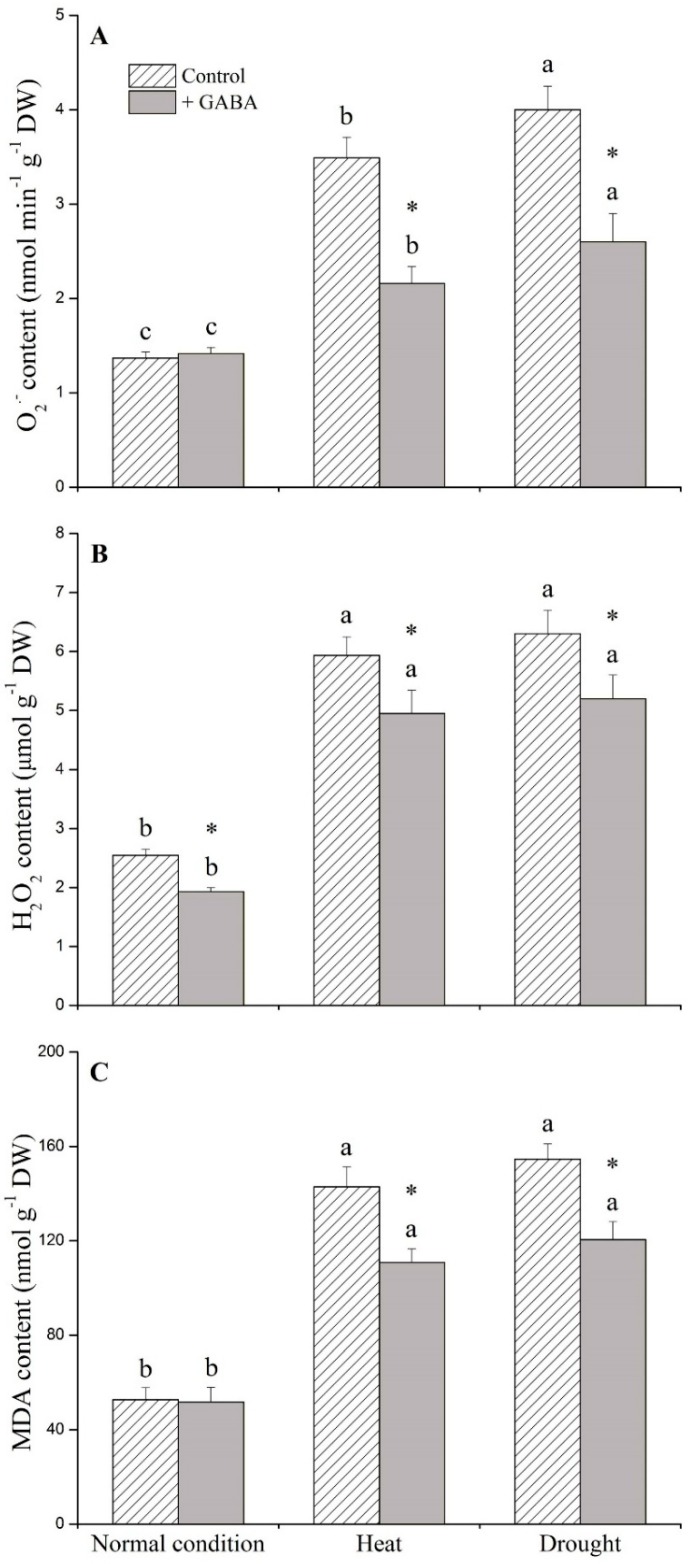
Effects of γ-aminobutyric acid (GABA) on (**A**) superoxide anion (O_2_^−^), (**B**) hydrogen peroxide (H_2_O_2_), and (**C**) malondialdehyde (MDA) content under normal, heat, and drought condition. Vertical bars indicate ±SE of mean (*n* = 4). Different letters above columns indicate significant differences for one particular treatment (control or +GABA) under different conditions; asterisk “*” indicates significant difference between control and +GABA treatment under a given condition.

Under non-stress conditions, exogenous GABA had no significant effects on any of the physiological parameters examined in this study (Figure 1, Figure 2 and Figure 3). GABA-treated plants had significantly higher turf quality (TQ) and relative water content (RWC), and lower electrolyte leakage (EL) than non-treated control plants under heat and drought stress (Figure 1A–C). GABA-treated plants had 34% and 38% higher chlorophyll content (Chl) than non-treated plants under heat and drought conditions, respectively (Figure 2A). The photochemical efficiency (Fv/Fm) ratio increased by 8% and 10% in GABA-treated plants compared to non-treated plants under heat and drought stress, respectively (Figure 2B). Drought and heat stress caused significant accumulation of O_2_^−^, H_2_O_2_, and malondialdehyde (MDA) in leaves but the application of GABA significantly reduced O_2_^−^, H_2_O_2_, and MDA content when plants were exposed to prolonged heat and drought conditions (Figure 3A–C).

### 2.2. Genes Associated with Signaling Transduction and Transcription Factors Affected by GABA

**Figure 4:**
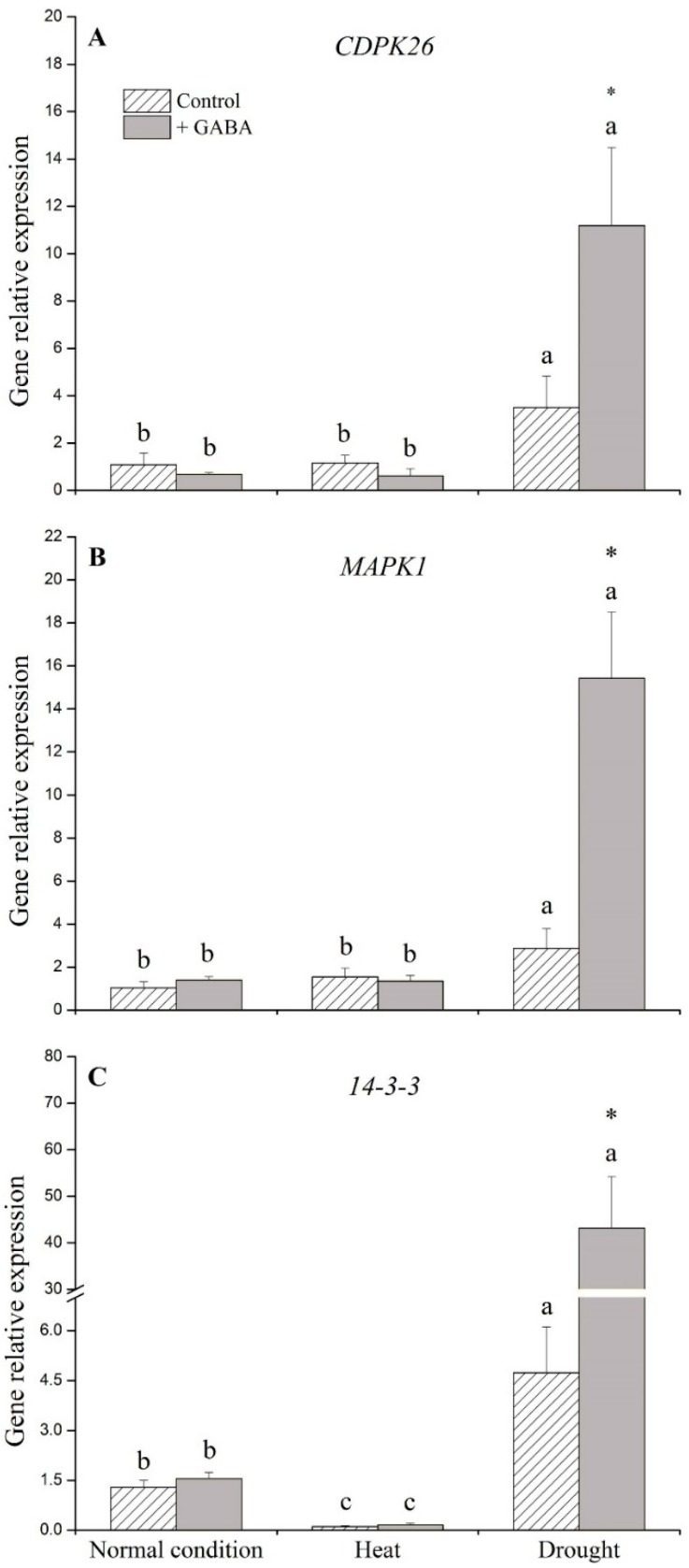
Effects of γ-aminobutyric acid (GABA) on genes’ expression of (**A**) *CDPK26*, (**B**) *MAPK1*, and (**C**) *14-3-3* under normal, heat, and drought conditions. Vertical bars indicate ±SE of mean (*n* = 4). Different letters above columns indicate significant differences for one particular treatment (control or +GABA) under different conditions; asterisk “*” indicates significant difference between control and +GABA treatment under a given condition.

**Figure 5:**
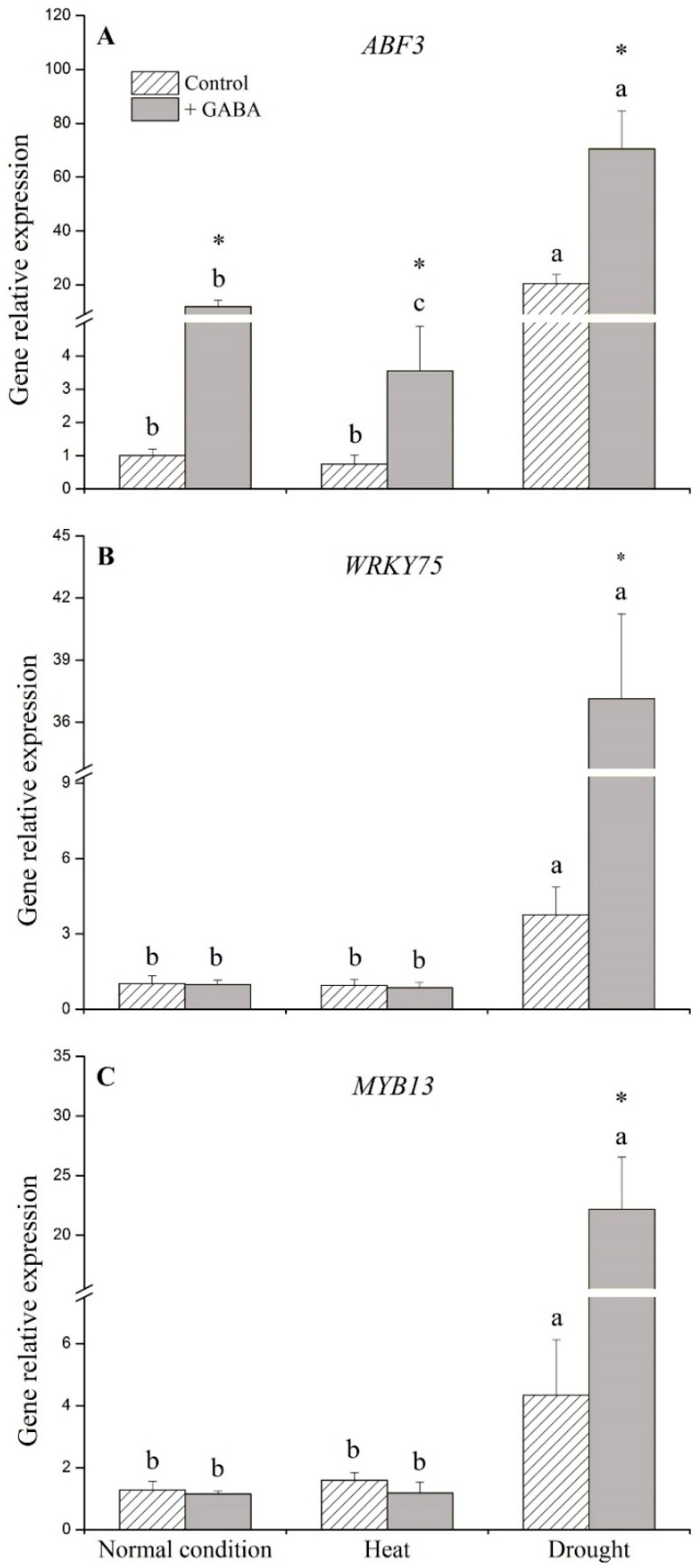
Effects of γ-aminobutyric acid (GABA) on genes’ expression of (**A**) *ABF3*, (**B**) *WRKY75*, and (**C**) *MYB13* transcription factors under normal, heat, and drought conditions. Vertical bars indicate ±SE of mean (*n* = 4). Different letters above columns indicate significant differences for one particular treatment (control or +GABA) under different conditions; asterisk “*” indicates significant difference between control and +GABA treatment under a given condition.

Transcript levels of *CDPK26* and *MAPK1* were not significantly affected by exogenous application of GABA under non-stress control and heat conditions (Figure 4A,B). Under drought stress, GABA application resulted in 3-, 5-, and 29-fold increases in the transcript levels of *CDPK26*, *MAPK1*, and *14-3-3*, respectively, compared to the non-treated control (Figure 4A–C). The transcript levels of *ABF3*, one important member of the *bZIP* family, increased 11- and 5-fold due to GABA application under non-stress conditions and heat stress, respectively. The transcript level of *ABF3*, *WRKY75*, or *MYB13* in GABA-treated plants was 3-, 10-, and 5-fold higher than those of non-treated plants under drought stress, respectively (Figure 5A–C).

### 2.3. Genes Associated with Antioxidant Enzymes Affected by GABA

**Figure 6:**
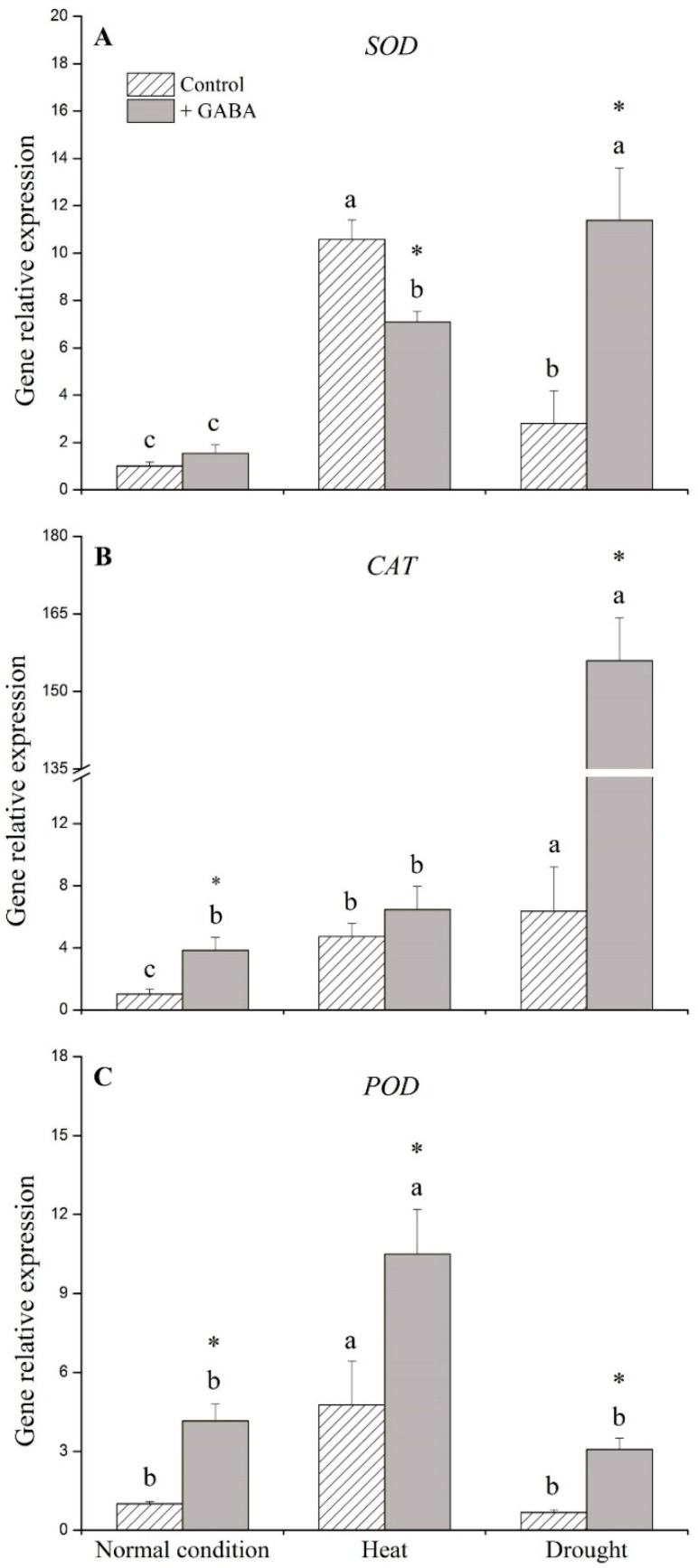
Effects of γ-aminobutyric acid (GABA) on genes’ expression of (**A**) *superoxide dismutase* (*SOD*), (**B**) *catalse (CAT*), and (**C**) *peroxidase* (*POD*) under normal, heat, and drought conditions. Vertical bars indicate ±SE of mean (*n* = 4). Different letters above columns indicate significant differences for one particular treatment (control or +GABA) under different conditions; asterisk “*” indicates significant difference between control and +GABA treatment under a given condition.

**Figure 7:**
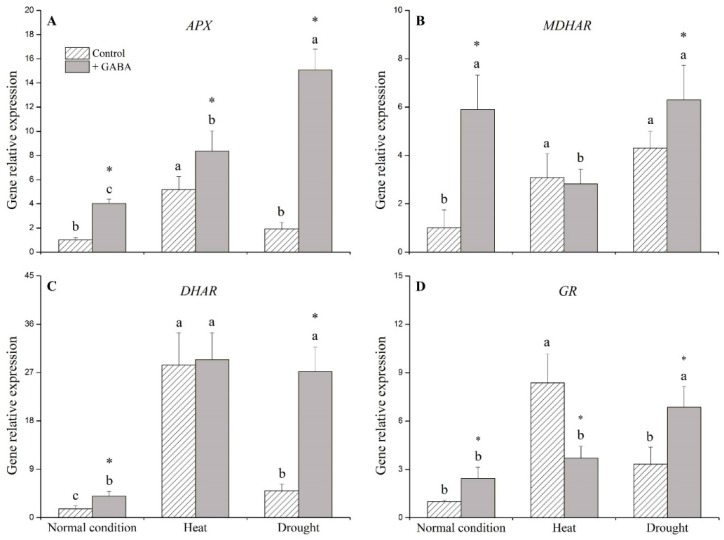
Effects of γ-aminobutyric acid (GABA) on genes’ expression of (**A**) ascorbate peroxidase (*APX*), (**B**) monodehydroascorbate reductase (*MDHAR*), (**C**) dehydroascorbate reductase (*DHAR*), and (**D**) glutathione reductase (*GR*) under normal, heat, and drought conditions. Vertical bars indicate ±SE of mean (*n* = 4). Different letters above columns indicate significant differences for one particular treatment (control or +GABA) under different conditions; asterisk “*” indicates significant difference between control and +GABA treatment under a given condition.

Under the non-stress control condition, exogenous application of GABA significantly up-regulated *catalse* (*CAT*) and *peroxidase* (*POD*), but did not affect *superoxide dismutase* (*SOD*) (Figure 6). *SOD* was down-regulated while *POD* was up-regulated by the application of GABA under heat stress (Figure 6A,C). Under drought stress, *SOD*, *CAT*, and *POD* levels of GABA-treated plants were 4-, 25-, and 5-fold higher than those of non-treated plants, respectively (Figure 6A–C). Exogenous application of GABA significantly up-regulated ascorbate peroxidase (*APX*), monodehydroascorbate reductase (*MDHAR*), dehydroascorbate reductase (*DHAR*), and glutathione reductase (*GR*) under the non-stress control condition (Figure 7A–D). Under heat stress, the application of GABA resulted in the up-regulation of *APX* and down-regulation of *GR* (Figure 7A,D). When plants were subjected to drought stress, GABA-treated plants showed significantly higher expression of *APX*, *MDHAR*, *DHAR*, and *GR* than non-treated plants (Figure 7A–D).

### 2.4. Genes Associated with Stress-Related Proteins Affected by GABA

**Figure 8:**
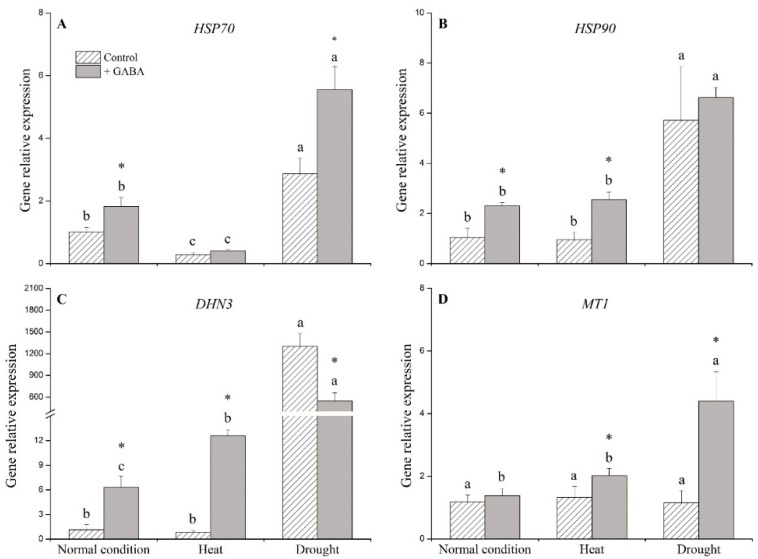
Effects of γ-aminobutyric acid (GABA) on genes’ expression of (**A**) heat shock protein 70 (*HSP70*), (**B**) heat shock protein 90 (*HSP90*), (**C**) dehydrin 3 (*DHN3*), and (**D**) metallothionein 1 (*MT1*) under normal, heat, and drought conditions. Vertical bars indicate ±SE of mean (*n* = 4). Different letters above columns indicate significant differences for one particular treatment (control or +GABA) under different conditions; asterisk “*” indicates significant difference between control and +GABA treatment under a given condition.

GABA-treated plants had significantly higher expression levels of heat shock protein 70 (*HSP70*), heat shock protein 90 (*HSP90*), and dehydrin 3 (*DHN3*) than non-treated plants under non-stress control condition (Figure 8A–C). Exogenous application of GABA up-regulated *HSP90*, *DHN3*, and *MT1* under heat stress (Figure 8B–D). Under drought stress, GABA treatment had 2- and 4-fold higher *HSP70* and *MT1* than under non-treated control, respectively (Figure 8A,D). The application of GABA down-regulated *DHN3* under drought stress (Figure 8C).

**Figure 9:**
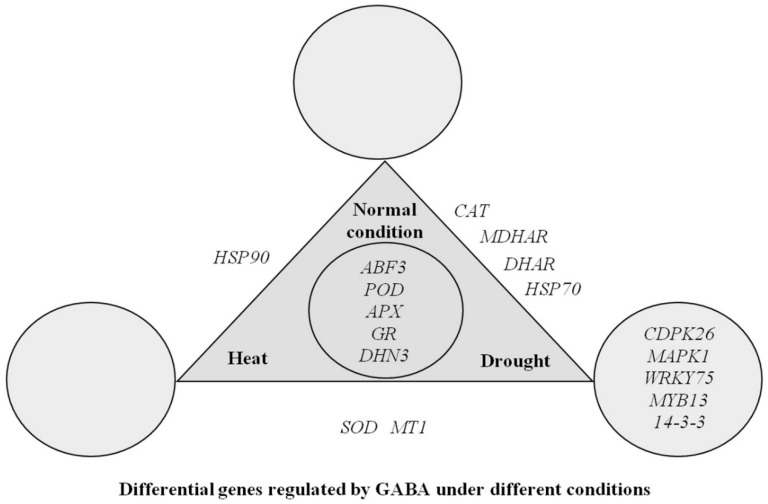
Differential expression of genes regulated by exogenous application of γ-aminobutyric acid (GABA) under different conditions.

Figure 9 summarizes differentially expressed genes that are regulated by exogenous application of GABA under non-stress, heat, or drought conditions. *ABF3*, *POD*, *APX*, *GR*, and *DHN3* were commonly regulated by exogenous application of GABA under all three conditions. *HSP90* was only regulated by GABA under non-stress and heat conditions. GABA regulated *CAT*, *MDHAR*, *DHAR*, and *HSP70* under non-stress and drought conditions. *SOD* and *MT1* were differentially regulated by GABA both under heat and drought. Exogenous GABA up-regulated *CDPK26*, *MAPK1*, *WRKY75*, *MYB13*, and *14-3-3* only under drought stress (Figure 9).

## 3. Discussion

As more research is being conducted regarding how GABA may be involved in regulating plant tolerance to abiotic and biotic stresses, there are increasing amounts of findings supporting its importance in this role [17,18,28]. In the current study, GABA had no significant effects on the physiological traits but heat and drought tolerance of creeping bentgrass was significantly improved by exogenous application of GABA, as demonstrated by increased leaf water content and cell membrane stability, less oxidative damage, and increased chlorophyll content during prolonged periods (20 d) of heat and drought stress. GABA-induced physiological effects related to improved heat and drought tolerance were in agreement with previous studies in rice and perennial ryegrass (*Lolium perenne*) [17,29]. Despite the strong efforts of previous research contributions, GABA-responsive genes and associated metabolic pathways that may be correlated with improved heat or drought tolerance in plants are not well-documented. Those genes and associated regulatory pathways related to heat and drought stress tolerance are discussed below in detail.

Ca^2+^-dependent protein kinase (CDPK) is known to initiate downstream signaling, leading to changes in transcript level of functional genes, which is an important part of the calcium messenger system [30,31,32]. Mitogen-activated protein kinase (MAPK) also has signaling roles in regulating cellular changes to extracellular stimuli, thereby controlling stress defense responses in plants [33,34]. Additionally, *14-3-3* protein has positive regulatory roles in the metabolism of carbon-nitrogen and cellular signaling such as CDPK, depending on the level of phosphorylation between *14-3-3* protein and target proteins in plants during a range of stress conditions [35,36,37]. It has been reported that the overexpression of the *Arabidopsis 14-3-3* gene *GF14λ* led to a stay-green phenotype and improved drought tolerance in cotton (*Gossypium spp*), as demonstrated by decreased wilting and a higher rate of photosynthesis under water deficit [38]. The *Arabidopsis* transformed with the tomato *14-3-3* gene *TFT7* exhibited enhanced tolerance to salt stress via activation of ascorbate peroxidase, resulting in the alleviation of salt-induced oxidative stress [39]. These previous studies, together with our current findings, suggest that GABA-upregulated *CDPK26*, *MAPK1*, and *14-3-3* expression levels could be associated with enhanced drought tolerance in creeping bentgrass through alleviating drought-induced leaf senescence and oxidative damage, since GABA-treated creeping bentgrass exhibited significantly higher chlorophyll content and Fv/Fm as well as lower ROS accumulation than untreated plants in response to drought stress. CDPK and MAPK involves the regulation of transcription factors such as *bZIP*, *WRKY*, and *MYB*, which act as regulators of downstream stress-related genes associated with acquired tolerance to abiotic stress in plants [10,32,40]. Transgenic rice seedlings overexpressing *WRKY11* exhibited significantly improved heat and drought tolerance [41]. The findings of Kim et al. [42] and Zhang et al. [43] confirmed that the overexpression of a *bZIP* factor could significantly enhance heat and drought tolerance of *Arabidopsis* seedling. The study of Oh et al. [44] also found that rice transformed with an *Arabidopsis ABF3* gene demonstrated significantly increased drought tolerance without displaying stunted growth. The results of current study demonstrated that exogenously applied GABA up-regulated three transcription factors (*ABF3*, *WRKY75*, and *MYB13*) under drought stress, indicating its positive association with drought tolerance in creeping bentgrass. Furthermore, the up-regulation of *ABF3* may contribute to GABA-induced heat tolerance in creeping bentgrass.

During exposure to prolonged heat and drought stress, one of the greatest contributors to plant tissue damage is overproduction of ROS [3,45]. ROS causes a variety of deleterious effects that compromise turf quality, such as lipid peroxidation, damage to nucleic acids, protein degradation, and activation of programmed cell death [46]. Superoxide dismutase (SOD) is a key enzyme that catalyzes the conversion of O_2_^−^ to H_2_O_2_, while catalse (CAT) and peroxidase (POD) are mainly responsible for scavenging H_2_O_2_ in plant cells [47]. In addition, the up-regulation of genes encoding enzymes involved in the ascorbate-glutathione (AsA-GSH) cycle led to detoxification of oxidative damages and was beneficial to plants under abiotic stresses [48]. It has been found that GABA-induced chilling and drought tolerance were regulated through activation of antioxidant enzyme activities to maintain cellular redox homeostasis in fruits of peach (*Amygdalus persica*) and leaves of perennial ryegrass [29,49]. Our previous study also found that foliar application of GABA could significantly enhance SOD, POD, APX, DHAR, and GR activities, contributing to the alleviation of oxidative damage in creeping bentgrass under heat stress [50]. In this study, significantly lower O_2_^−^, H_2_O_2_, and MDA contents were observed in GABA-treated plants compared to those in non-treated plants under heat and drought stress with the enhancement of gene transcript levels encoding antioxidant enzymes, suggesting that GABA-regulated antioxidant defense could be partly responsible for improved heat and drought tolerance in creeping bentgrass. Our data further confirmed that GABA was involved in the modification of antioxidant defense in creeping bentgrass at molecular level.

The accumulation of defense proteins such as HSPs, DHNs, and MT is another determinant of plant adaptation to abiotic stress [9,51,52,53]. HSPs, known as molecular chaperones, function in assisting protein folding and translocation, correcting structures of macromolecules, and preventing protein aggregation [54]. A large number of studies have confirmed that HSPs are particularly important for plant survival under multiple abiotic stresses including heat and drought stress [55,56,57]. Current results showed that *HSP70* was up-regulated by GABA under drought stress. In contrast, exogenous GABA only increased the expression of *HSP90* in leaves exposed to heat stress, indicating that the regulatory roles of GABA for activation of HSPs may differ in response to heat and drought. In addition, DHN*s* are known to play important roles in the stabilization of cellular membranes and osmotic adjustment when plants are exposed to drought stress [52,58,59]. The expression of DHNs proteins was also positively associated with enhanced heat tolerance in sugarcane (*Saccharum officinarum*) [60,61]. In the present study, GABA-treated plants exhibited significantly higher *DHN3* level than non-treated plants during heat stress, indicating GABA-enhanced expression of *DHN3* could contribute to improved heat tolerance associated with the maintenance of better membranes stability and water status (higher RWC in leaf) in creeping bentgrass. Similarly, MT has been found to account for metal ion homeostasis and neutralization of O_2_^−^ and hydroxyl radicals against oxidative damage [62,63]. It has been confirmed that higher MT accumulation or overexpression of genes encoding MT could improve drought tolerance of white clover (*Trifolium repens*) and rice [9,64]. The exogenous application of GABA along with the up-regulation of *MT1* transcript level suggests that *MT1* may be involved in GABA-improved heat and drought tolerance through scavenging O_2_^−^ and hydroxyl radicals against oxidative damage in creeping bentgrass.

## 4. Materials and Methods

### 4.1. Plant Materials and Growth Conditions

Creeping bentgrass (cv. “Penncross”) sods (6 cm in diameter) were transplanted into a plastic container which was filled with fritted clay (Profile Products, Deerfield, IL, USA), and grown in a greenhouse (Horticultural Farm II at Rutgers University, North Brunswick, NJ, USA). A total of 12 containers with 8 sod plugs in each container were used for the following experiment. The environmental conditions in the greenhouse included average temperature of 23/16 °C (day/night) and 790 μmol m^−2^ s^−1^ photosynthetically active radiation (PAR) with the use of supplemental sodium lights on overcast days. Half-strength Hoagland’s nutrient [65] was used for fertilizing plants once a week. For maintenance of a canopy height of approximately 4 cm, plants were trimmed twice a week and grew for 2 months in the greenhouse. Plants were then moved to a controlled-environment growth chamber (Environmental Growth Chamber, Chagrin Falls, OH, USA) for one week to allow plants to acclimate to the growth chamber conditions prior to exposure to heat or drought stress. The growth chamber conditions were set to 23/18 °C (day/night), 70% relative humidity, 750 µmol m^−2^ s^−1^ PAR, and a 12 h photoperiod.

### 4.2. GABA and Stress Treatments

For GABA treatment, 10 mLof 0.5 mM GABA solution was sprayed on leaves of four sod plugs in each container. The four untreated control sods were sprayed with water. Both GABA and water control were applied three times at 2-d intervals before being exposed to non-stress control, heat stress, or drought stress conditions. The concentration of GABA utilized in this study was selected as the optimal effective dose based on a preliminary experiment under heat and drought conditions. Both GABA and untreated control had four replicates in four containers. GABA-treated plants and untreated control plants were subjected to the following three treatments: (1) Non-stress control: soil volumetric water content (SWC) was maintained at the pot capacity (27%) at 23/18 °C (day/night) for 20 d; (2) Heat stress: plants were maintained as well-watered but were subjected to high temperature (35/30 °C, day/night) for 20 d; and (3) Drought stress: plants received no irrigation and were maintained at 23/18 °C (day/night) for 20 d until SWC declined to 7%. A time domain reflectometer (TDR) (Trase Soil Moisture Equipment, Santa Barbara, CA, USA) was used to monitor SWC. Each stress treatment and the non-stress control had four replicates in four containers. The experiment was arranged in a split-plot design with stress treatments (heat and drought) as the main plots and GABA treatments as the sub-plots. Each stress treatment was repeated in four growth chambers. A total of eight chambers were used with four chambers controlled at 35/30 °C (day/night) for heat stress and four chambers controlled at 23/18 °C for the non-stress control and drought treatments. The GABA-treated plants and untreated control had four replicates in four containers which were placed in four growth chambers.

### 4.3. Physiological Analysis

Several commonly-used stress indicators were examined to evaluate the physiological effects of GABA. Turf quality (TQ) was visually rated using a scale of 1 to 9 based on color, density, texture, and uniformity of the grass [66]. Leaf relative water content (RWC) was calculated using the formula RWC (%) = [(FW–DW)/(TW–DW)] × 100 where FW, TW, and DW stand for fresh leaves, turgid weight, and dry weight, respectively [67]. For the determination of leaf electrolyte leakage (EL), 0.1 g of fresh leaves were immersed in 35 mLof deionized water after being rinsed three times with deionized water. Leaf samples were shaken for 24 h to measure initial conductivity (C_initial_), autoclaved at 120 °C for 20 min and maximum conductance (C_max_) of killed leaves was measured using a conductivity meter (YSI Model 32, Yellow Spring, OH, USA). EL was calculated according to the percentage of C_initial_/C_max_ [68]. The leaf chlorophyll (Chl) content was analyzed using the method of Arnon [69] and the fluorescence meter (Dynamax, Houston, TX, USA) was used for the measurement of photochemical efficiency (Fv/Fm) of leaf. In order to determine whether GABA may affect oxidative damages induced by heat and drought stress, the content of superoxide anion (O_2_^−^) [70] and hydrogen peroxide (H_2_O_2_) [71], as well as the lipid peroxidation product, malondialdehyde (MDA) [72], was quantified. The procedure and reagents for measurement of O_2_^−^, H_2_O_2_, and MDA have been clearly described in our previous study [50].

### 4.4. Total RNA Extraction and qRT-PCR Analysis

To determine the effects of GABA on changes of selected gene transcripts and transcriptional factors involved in stress protection, transcript levels of genes were analyzed using real-time quantitative polymerase chain reaction (qRT-PCR). The RNeasy Mini Kit (Qiagen, Duesseldorf, Germany) was used for total RNA extraction according to the manufacturer’s instructions. The RNA was then reverse-transcribed to get cDNA using the Revert Aid First Strand cDNA Synthesis Kit (Fermentas, Lithuania). All primer sequences used in this study are shown in Table 1. The PCR conditions were as follows: 95 °C for 5 min and then 95 °C for 15 s (40 repeats of denaturation), annealing at 60 °C for 45 s, and heating of the amplicon from 60 to 95 °C. The formula 2^−ΔΔCt^ described by Xia et al. [73] was employed for calculating the transcript levels of all genes, and *ACT2* was used as reference gene.

### 4.5. Statistical Analysis

The statistical analysis system (SAS) (SAS Institute, North Carolina, USA, 2008) was used to determine differences between means of treatment effects on all measured parameters. Significant differences between treatment means were tested using Fisher’s protected least significant difference test (LSD) at *p* = 0.05.

## 5. Conclusions

In conclusion, our results demonstrated that exogenous application of GABA could significantly improve heat and drought tolerance in creeping bentgrass, as reflected by less leaf water deficit and oxidative damage, higher cell membrane stability, and higher chlorophyll content under prolonged heat and drought stress. Exogenous application of GABA up-regulated some stress-related genes including *ABF3*, *POD*, *APX*, *HSP90*, *DHN3*, and *MT1* under heat stress, and also induced the expression of *CDPK26*, *MAPK1*, *ABF3*, *WRKY75*, *MYB13*, and *HSP70*, *MT1*, *14-3-3*, and genes encoding antioxidant enzymes (*SOD*, *CAT*, *POD*, *APX*, *MDHAR*, *DHAR*, and *GR*) under drought stress. It is noteworthy that four genes *ABF3*, *POD*, *APX*, and *MT1* are commonly upregulated by GABA under drought and heat stress, indicating that these genes could play extremely important roles in GABA-induced drought and heat tolerance in creeping bentgrass. The results also suggest that *ABF3*, *POD*, *APX*, and *MT1* could be key candidate genes for improving drought and heat tolerance of creeping bentgrass through transgenic approach. These findings indicate that GABA enhanced heat and drought tolerance as a result of its effects on the expression of genes and associated pathways for stress signaling and protection. In the future, mechanisms of how GABA may regulate those above-mentioned genes involved in heat and drought tolerance deserve further investigation.

## Figures and Tables

**Table 1:** Primer sequences used in qRT-PCR and their corresponding GeneBank accession numbers of the analyzed genes.

Target Gene	Accession No.	Forward Primer (5′–3′)	Reverse Primer (5′–3′)
*SOD*	DV867103	CACTGGACCTCACTTCAAC	GTAGCAACACCATCCACTC
*POD*	DV867327	CTTCGACAACGCCTACTAC	TTTGCCCATGTTCACCA
*CAT*	DY543619	TTGCCAATAAGAGGGAGAATG	CGAAGCCGAGCATGTAAG
*APX*	GR281667	AGGACATTGTTGCCCTTTC	GCTCCGTGAAGTAAGAGTTG
*DHAR*	DV853556	GAAAGGTGCCTGTGTTTAATG	GTGATGGAGTTGGGTACTTC
*GR*	AB277097	GATGGAGGCTACTTGCTTTG	GCTAAGACCCACGACAGATA
*MDHAR*	DV865007	CCATGAAGCTCTACAACGAG	GTAGAAGTAGGGCAGGTAGT
*HSP70*	DV860338.1	CCTGCCCAATTTGCATTACC	CAGACGGAGAAGCAACTGAA
*HSP90*	GR280041.1	CCACCCATACTCACCTGTCACG	CAAGGAGAAGTTTGAAGGGCTATG
*DHN3*	FE527922.1	CATGGCGTCTACTGCTTGTA	CAGAGGACTTGAACCCAGATAC
*MT1*	DV865927.1	TCTCCAAGCTCATCTTCTTCTCATT	TTCGTCCAGGTCAGGGTACATC
*WRKY75*	DV867719.1	TGGTGGTGACGACATACGAGG	GGTTGGTAAAGGTTGAGGAGGTG
*MYB13*	GR279830.1	CATTCAGTTTACCCGAGTGCG	CATAAAACATGACCCATCACAGCT
*ABF3*	DV862003.1	ATCTGCCTGCGGAGGACACT	TGAAGCATCGGAACAGTGGC
*CDPK26*	GR281936.1	ATCCAGGCTGCTCACTCCGTA	AACCAACGCAGGGTAGGATTTC
*MAPK1*	DV866362.1	AGCTGGCCCTGCATGGATAA	CAGGACAATGTTCAGATGGAGGC
*14-3-3*	DV866921.1	TCATGGACAAGATCAAGGAGAAG	CAAACACCCAAGTGAGCTAAAC
*ACT2*	DY543529	CCTTTTCCAGCCATCTTTCA	GAGGTCCTTCCTGATATCCA
